# Research progress of silicon-based anode materials for lithium-ion batteries

**DOI:** 10.1039/d5ra01268f

**Published:** 2025-04-07

**Authors:** Zhenjun Zhang, Yilong Wu, Zuxue Mo, Xiaoxu Lei, Xuerui Xie, Xiangyong Xue, Haiqing Qin, Haowen Jiang

**Affiliations:** a Guangxi Key Laboratory of Superhard Material, National Engineering Research Center for Special Mineral Material, Guangxi Technology Innovation Center for Special Mineral Material, China Nonferrous Metal (Guilin) Geology And Mining Co., Ltd Guilin 541004 P. R. China 1849095151@qq.com

## Abstract

In recent years, with the rapid development of fields such as portable electronic devices, electric vehicles, and energy storage systems, the performance requirements for lithium-ion batteries have been continuously rising. Among the numerous key components of lithium-ion batteries, the performance of the anode materials plays a crucial role, as it is directly related to core indicators such as the energy density, cycle life, and safety of the batteries. Among them, silicon-based anode materials have stood out among many anode materials by virtue of their extremely high theoretical specific capacity, becoming one of the hot research directions in the field of lithium-ion battery anode materials at present. However, silicon-based anode materials have problems such as severe volume expansion, poor electrical conductivity, low initial coulombic efficiency, and unstable solid electrolyte interphase during the charging and discharging process, which limit their wide application and urgently require the seeking of new solutions. This paper comprehensively and in-depth introduces the research progress of silicon-based anode materials for lithium-ion batteries in recent years, focusing on the failure mechanisms and modification methods of silicon-based anodes, and provides effective solutions to the severe challenges faced in the commercialization process of silicon-based anodes.

## Abstract 2

In recent years, with the rapid development of fields such as portable electronic devices, electric vehicles, and energy storage systems, the performance requirements for lithium-ion batteries have been continuously rising.

## Graphical abstract

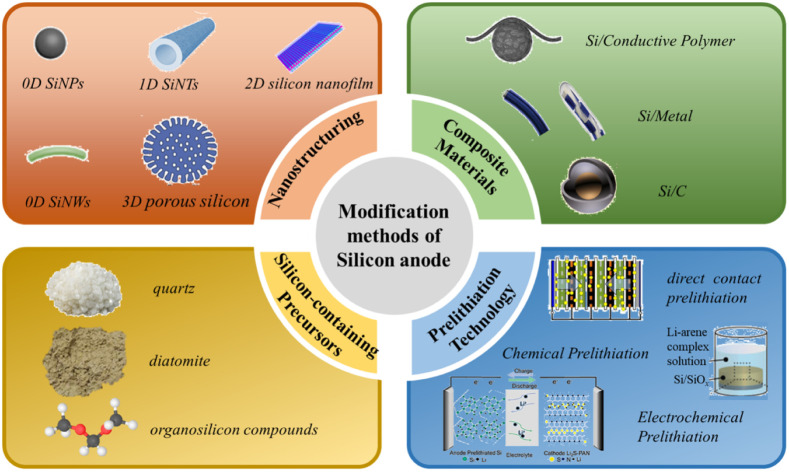

## 1 Introduction

Lithium-ion batteries, as an efficient energy storage device, play a crucial role in various fields of modern society. From portable electronic devices to electric vehicles and then to large-scale energy storage systems, the performance and application range of lithium-ion batteries have been continuously expanded. In the development process of lithium-ion batteries, searching for high-capacity and high-performance anode materials has always been one of the research focuses. Silicon-based anodes have gradually become a research hotspot due to their unique advantages. Since its commercialization in the 1990s, the lithium-ion battery has undergone multiple technological innovations. Its advantages such as high energy density, long cycle life, and low self-discharge rate^1–3^ have made it the preferred energy storage technology in fields such as consumer electronics, electric vehicles, and renewable energy storage. The development of lithium-ion batteries has not only promoted the miniaturization and intellectualization of electronic devices but also provided strong support for the popularization of electric vehicles and the large-scale application of renewable energy. With the continuous increase in the requirements for the energy density and performance of lithium-ion batteries, the traditional graphite anode is gradually unable to meet the demand. Silicon has an extremely high theoretical specific capacity (about 4200 mA h g^−1^),^4,5^ which is more than ten times that of graphite. However, silicon undergoes a huge volume change (about 300%),^6,7^ during the charging and discharging process, resulting in the destruction of the electrode structure and rapid capacity decay. Optimizing the silicon structure and compounding silicon with other materials can alleviate the volume expansion problem of silicon to a certain extent,^8,9^ effectively improve the cycle performance and rate performance of the silicon anode, and are expected to achieve a high-performance lithium-ion battery anode.

## 2. Structure and principle of lithium-ion batteries

**Fig. 1:**
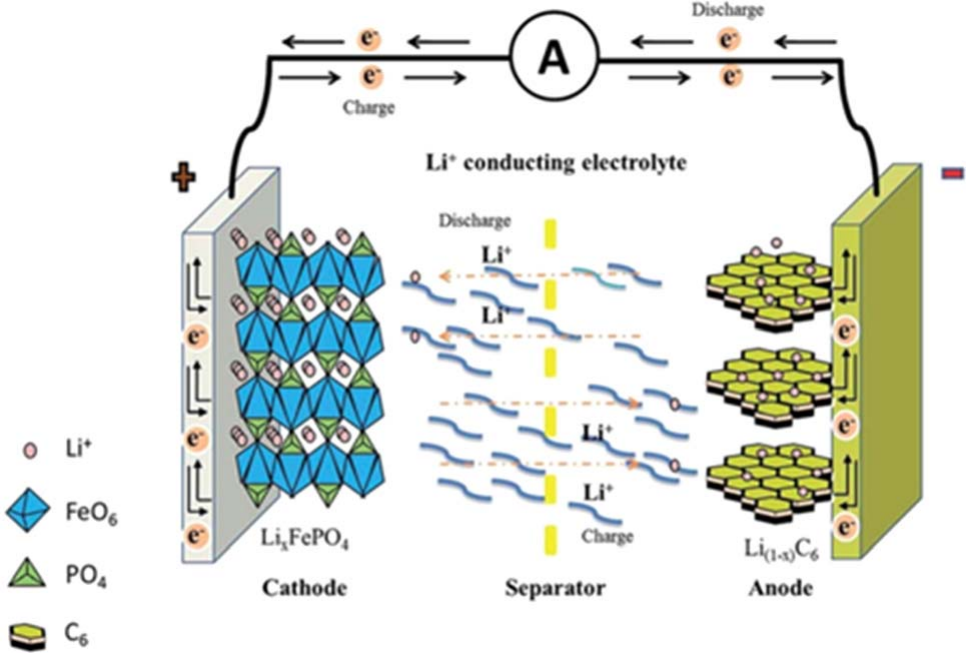
Schematic diagram of the working principle of lithium-ion batteries.^10^

A lithium-ion battery mainly consists of five parts: a cathode, an anode, a separator, an electrolyte, and a casing. Its working principle is based on the continuous intercalation and deintercalation of lithium ions, which are combined with electrons at the same time. Taking a battery system composed of LiFePO_4_ (lithium iron phosphate) as the cathode and graphite as the anode as an example (Fig. 1): the charging and discharging process is achieved through the migration of lithium ions between the cathode and the anode.

During charging, an external power source is connected to the battery. An oxidation reaction occurs at the positive electrode, and lithium ions are extracted from LiFePO_4_, releasing electrons. The electrons flow to the negative electrode through the external circuit, while the lithium ions migrate through the electrolyte and across the separator to the negative electrode. At the negative electrode, the lithium ions are intercalated between the graphite layers to form a lithium-carbon compound (Li_*x*_C_6_).

1 LiFePO_4_ → Li_1−*x*_FePO_4_ + *x*Li^+^+*x*e^−^

2 6C + *x*Li^+^ → Li_*x*_C_6_

During discharging, the battery is connected to an external load. Electrons flow from the negative electrode to the positive electrode through the external circuit. At the same time, lithium ions are extracted from the negative electrode and return to the positive electrode through the electrolyte and the separator. At the positive electrode, the lithium ions are intercalated into the LiFePO_4_ structure and combine with electrons, undergoing a reduction reaction.

3 Li_1−*x*_FePO_4_ + *x*Li^+^ + *x*e^−^ → LiFePO_4_

4 Li_*x*_C_6_ → 6C + *x*Li^+^ + *x*e^−^

During this process, the LiFePO_4_ of the positive electrode material loses lithium ions during charging and reabsorbs them during discharging. Meanwhile, the graphite of the negative electrode material absorbs lithium ions during charging and releases them during discharging. These chemical reactions enable the migration of lithium ions between the positive and negative electrodes, thus realizing the charging and discharging of the battery and fulfilling the energy storage and release of the battery.

During the intercalation and deintercalation of lithium ions, the intercalation and deintercalation of an equivalent amount of electrons accompany lithium ions (customarily, the terms intercalation or deintercalation are used for the positive electrode, while insertion or extraction are used for the negative electrode). It is like a rocking chair movement of lithium ions back and forth between the positive and negative electrodes. Therefore, the lithium-ion battery is vividly called a rocking chair battery.^11^

The cathode of a lithium-ion battery has a relatively high potential and is often a lithium-intercalated transition metal oxide or a polyanionic compound, such as lithium cobalt oxide^12,13^ (LiCoO_2_), lithium manganate^14,15^ (LiMn_2_O_4_), ternary materials^16,17^ (such as lithium nickel cobalt manganese oxide LiNi_1−*x*−*y*_Co_*x*_Mn_*y*_O_2_), lithium iron phosphate^18^ (LiFePO_4_), *etc.*; The anode is usually a carbon material, such as graphite and non-graphitized carbon. The electrolyte is mainly a non-aqueous solution composed of an organic mixed solvent and a lithium salt. The solvent is mostly an organic solvent like carbonate, and the lithium salt is mostly a monovalent polyanionic lithium salt, such as lithium hexafluorophosphate (LiPF_6_). The separator is generally a microporous film of polyethylene or polypropylene, which functions to separate the cathode and anode materials, prevent electrons from passing through to cause a short circuit, and allow ions in the electrolyte to pass through.

## 3 Introduction to anode materials of lithium-ion batteries

### 3.1 Intercalation reaction type anode

In lithium-ion batteries, the intercalation reaction type anode refers to the anode material that realizes charge storage and release through the intercalation and deintercalation of lithium ions between the material layers. Common intercalation reaction type anode materials include graphite, metal oxides, *etc.* Graphite is currently the most commercialized anode material for lithium-ion batteries. It has a layered structure, and Li^+^ can be relatively easily intercalated between the graphite layers and combined with 6 carbon atoms to form LiC_6_,^19^ with a corresponding theoretical specific capacity of 372 mA h g^−1^. This intercalation reaction is relatively stable, endowing the graphite anode with good cycling performance. However, the discharge platform of LiC_6_ is relatively low. This low discharge platform restricts the output voltage of the battery. For some electrical equipment or application scenarios with high voltage requirements, it may not be able to meet their normal operating needs. The lithium intercalation potential of graphite is close to that of the lithium anode. During the charging process, it is prone to causing the deposition and precipitation of metallic lithium on the surface of the anode. Lithium deposition not only leads to a rapid decline in the battery capacity but may also pierce the separator, causing an internal short circuit in the battery. At the same time, the graphite anode undergoes various side reactions with the electrolyte during the charging and discharging processes. These side reactions result in the formation of an unstable solid electrolyte interface (SEI) film on the anode surface, reducing the first coulombic efficiency of the battery.

Besides graphite-based materials, lithium titanate also belongs to the intercalation-reaction-type anode materials. Its lithium-storage mechanism is the transformation between spinel-structured lithium titanate (Li_4_Ti_5_O_12_) and rock-salt-structured lithium titanate (Li_7_Ti_5_O_12_).^20^ During the charging and discharging processes, it has a very small volume change, which endows the battery with a long cycle life.^21,22^ Its relatively high potential can avoid the generation of lithium dendrites and improve the safety of the battery. However, its specific capacity is relatively low, which limits the energy density of the battery to a certain extent. Moreover, its poor electronic conductivity will affect the rate performance of the battery.

In general, intercalation reaction-type anodes have important applications in lithium-ion batteries. However, continuous research and improvement are still needed to meet the demands for higher energy density, longer cycle life, and better rate performance.

### 3.2 Conversion-reaction-type anodes

Conversion-reaction-type anode materials for lithium-ion batteries refer to those that store lithium with a high specific capacity through the reversible displacement redox reaction between Li and transition-metal cations. These materials include metal oxides, metal sulfides, metal hydrides, metal nitrides, metal phosphides, metal fluorides, *etc.* The electrochemical reaction formula is as follows:

5 M_*a*_X_*b*_ + (*b*·*n*)Li^+^ + (*b*·*n*)e^−^ → *a*M + *b*Li_*n*_X

**Fig. 2:**
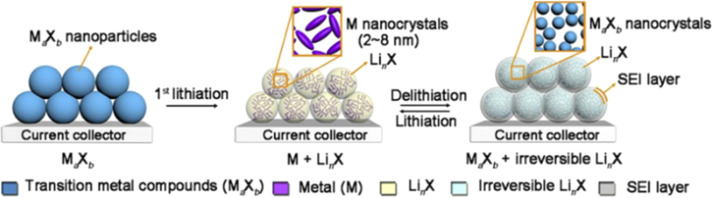
Schematic diagram of local chemical transformations of transition metal compounds during the conversion reaction process.^23^

As shown in Fig. 2, during the first lithiation of M_*a*_X_*b*_, M nanocrystalline particles and Li_*n*_X coated with a solid-electrolyte-interface (SEI) film are formed. The key to the reversibility of the subsequent conversion mechanism lies in the formation of highly electroactive M nanocrystalline particles to decompose the SEI layer on the Li_*n*_X matrix. Many conversion-reaction-type anodes (such as Fe_3_O_4_, FeS_2_, and MnO_2_) exist in natural forms (magnetite, pyrite, and pyrolusite), with relatively low production costs. Conversion-reaction-type anodes exhibit adjustable reaction potentials, which specifically depend on the ionic-bond strength between the transition-metal (M) cations and the anionic substances. Compared with the graphite anode with a low lithium-insertion potential, conversion-reaction-type anodes can ensure better battery safety by avoiding the problem of lithium-dendrite formation. They have higher specific capacities and better safety compared to intercalation-type materials. However, due to their inherently poor electronic and ionic conductivity, relatively large volume expansion (<200%), and continuous electrolyte decomposition, conversion-reaction-type anodes still face significant challenges.

### 3.3 Lithium metal anode

The lithium-metal anode has high conductivity and a high theoretical specific capacity (3860 mA h g^−1^, which is equivalent to ten times that of the graphite anode of commercial lithium-ion batteries).^24^ Moreover, it has the lowest anode potential and can be paired with the existing cathode systems of lithium-ion batteries, making it a highly promising anode material.

In the development history of lithium-metal anodes, early research can be traced back to the last century. In 1913, Gilbert N. Lewis first proposed the concept of a lithium-metal anode.^25^ Harris first discovered in 1958 that lithium metal could stably exist in non-aqueous solvents. During the 1960s and 1970s, primary batteries using lithium metal as the anode and non-aqueous solvent electrolytes were extensively studied. In the late 1980s, the commercial MoS_2_/Li battery developed by Moli Company had a greatly enhanced energy density and occupied a large market share. However, later, due to several fire and explosion incidents of lithium-ion batteries, a large-scale product recall was forced.^26^ Subsequent research showed that the cause of the fire and explosion of lithium-ion batteries was the growth of lithium dendrites during charging and discharging, which pierced the separator, leading to an internal short-circuit and then thermal runaway.^27^ In the 1990s, due to the inability to completely solve safety hazards such as the growth of lithium dendrites, scientists turned to study other anode materials. By the 21st century, as the specific capacity of common anodes for lithium-ion batteries approached their theoretical values, and with the proposal of new battery systems such as lithium-sulfur and lithium-air batteries, researchers had to return to the study of lithium-metal anodes. After 2010, the development of solid electrolytes such as polymers,^28,29^ oxides, and sulfides brought new directions to the research of lithium-metal anodes. Replacing traditional liquid electrolytes with solid electrolytes can effectively suppress the growth of lithium dendrites, reduce interfacial side reactions, and increase the energy density.^30^ At present, certain progress has been made in improving the ionic conductivity of solid electrolytes. However, the safety issues caused by the growth of lithium dendrites still pose a huge obstacle on the path to commercial applications.

### 3.4 Alloy-reaction-type anode

Alloy-reaction-type anode materials for lithium-ion batteries refer to metals and their alloys that can undergo alloying reactions with lithium. Some common metals that can alloy with lithium include tin (Sn), aluminum (Al), germanium (Ge), magnesium (Mg), calcium (Ca), silicon (Si), *etc.* The theoretical specific capacity and charge density of this type of anode material are generally higher than those of intercalation-type anode materials. Additionally, compared with the lithium–intercalation potential of graphite (<0.1 V *vs.* Li/Li^+^), the lithium-intercalation voltage of alloying anodes is slightly higher. This is beneficial for avoiding the growth of lithium dendrites and improving the safety of the battery.^31–34^ Meanwhile, their relatively low operating potential (∼0.3–0.6 V *vs.* Li/Li^+^) is also conducive to matching high-voltage cathode materials, thus enabling the development of lithium-ion batteries with higher energy density.

Tin and its oxides are among the materials in this type of anode that have been studied relatively early.^35,36^ The theoretical specific capacity of Sn^37^ is 990 mA h g^−1^,and that of SnO_2_ is 1494 mA h g^−1^, which are respectively more than 2 times and 4 times the theoretical capacity of graphite carbon.^38,39^ However, during the lithium-insertion and extraction processes, tin-based materials undergo phase transitions and alloying reactions, generating a huge volume expansion effect. This causes the material to be pulverized, its structure to be damaged, resulting in a sharp decline in capacity and poor cycling performance.^40^ In addition, as a metal oxide, SnO_2_ material has poor electronic conductivity, thus its rate performance is also unsatisfactory.

**Fig. 3:**
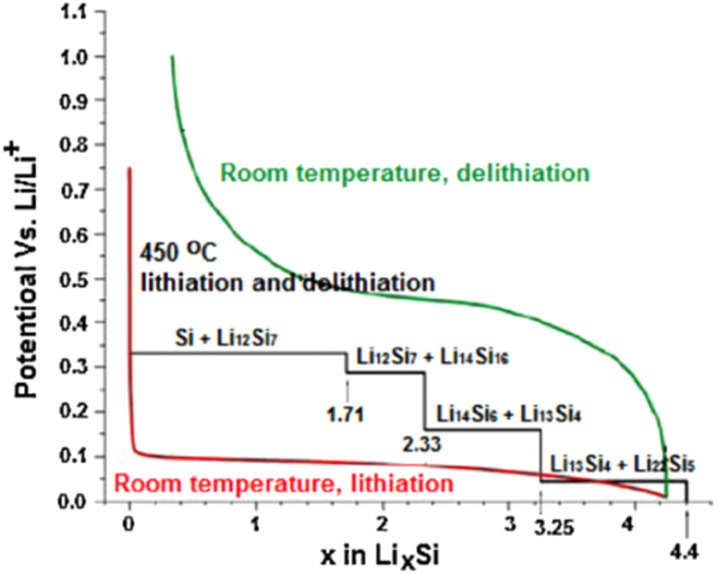
The delithiation and lithiation curves of silicon at room temperature and 450 °C.^41^

Silicon has received extensive attention due to its high capacity. As shown in Fig. 3, the electrochemical de-/intercalation curves of lithium in silicon at room temperature and high temperature are presented. The results show that lithium and silicon can form four intermediate-phase alloys, namely Li_12_Si_7_, Li_7_Si_3_, Li_13_Si_4_ and Li_22_Si_5_. Among them, the theoretical specific capacity corresponding to Li_22_Si_5_ is 4200 mA h g^−1^, which is 10 times higher than that of graphite. However, like tin-based materials, silicon-based materials undergo phase transitions and alloying reactions during the lithium-insertion and extraction processes, generating a huge volume-expansion effect. This causes the material to be pulverized and its structure to be damaged, leading to a sharp decline in capacity and poor cycling performance.^42^

In the system of alloy-reaction-type anode materials, silicon-based anodes have become highly promising research objects in this field due to their extremely high theoretical specific capacity of up to 4200 mA h g^−1^, far exceeding that of most similar materials. Compared with traditional intercalation-reaction-type anodes, their theoretical specific capacity shows a significant advantage, providing broad prospects for enhancing the energy density of batteries. In comparison with conversion-reaction-type anodes, silicon-based anodes have unique advantages in terms of lithium-intercalation voltage and compatibility with high-voltage cathode materials, and play a key role in constructing high-energy-density battery systems. However, in the process of moving towards large-scale practical applications, silicon-based anodes face numerous severe challenges, which makes the modification research on them an important topic in the current lithium-battery field. The following text will elaborate in detail on the inherent disadvantages that restrict the development of silicon-based anodes while they possess outstanding advantages, as well as the cutting-edge modification work carried out to address these issues.

## 4 Problems existing in silicon-based anode materials

**Fig. 4:**
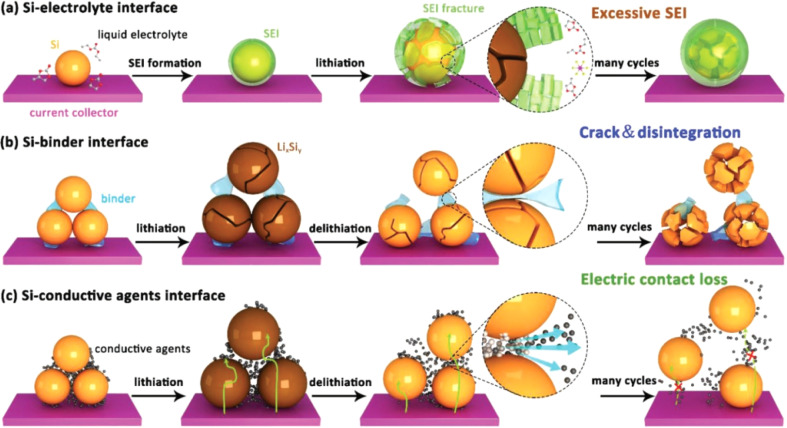
Various issues faced by silicon anodes: (a) schematic diagram of the failure process at the electrolyte interface; (b) schematic diagram of the failure process at the polymer binder interface; (c) schematic diagram of the failure process at the conductive additive interface.^52^

During the charging and discharging process, an alloying reaction occurs between silicon and lithium, causing a significant volume expansion of silicon. This repeated contraction and expansion can lead to the formation of cracks in the silicon-based anode material until it pulverizes.^43–48^ As a result, the contact between the electrode material and the current collector is disrupted, and the active material detaches from the electrode sheet, leading to a rapid decline in battery capacity.^49–51^ These problems severely limit the practical application of silicon-based anodes. The interfacial evolution patterns of Si can be classified, and the corresponding failure mechanisms can be briefly summarized into the following three types (Fig. 4).

### 4.1 Electrolyte-interface failure

During the first charge–discharge process of silicon, a reaction occurs between the electrode material and the electrolyte at the solid–liquid interface, forming a passivation layer covering the surface of the electrode material, namely the solid-electrolyte-interface (SEI) film. The SEI film provides specific channels for the intercalation and de-intercalation of lithium ions between the electrode and the electrolyte, facilitating the transmission of lithium ions. At the same time, it can protect the electrode material from being corroded by the electrolyte and extend the service life of the electrode. The formation and subsequent repair processes of the SEI film consume a certain amount of lithium ions, resulting in a reduction in the available lithium ions in the battery, thus reducing the battery capacity. During the battery cycling process, due to factors such as the volume change of the electrode material, the SEI film will break and reform, causing the film to continuously thicken.^53–56^ An overly thick SEI film will further impede the transmission of lithium ions and consume a large amount of electrolyte, affecting the battery performance.^57–62^

### 4.2 Polymer binder interface failure

During the charging and discharging process, silicon undergoes significant volume changes. This intense expansion and contraction exert enormous pressure on the interface between silicon and the binder. Since the binder cannot effectively withstand and adapt to this continuous volume change, the bonding strength at the interface gradually weakens. This not only affects the transmission efficiency of electrons and ions at the interface but also causes silicon particles to easily detach from the binder, thus destroying the structural integrity of the electrode.^63,64^ As the number of cycles increases, the problem of interface instability becomes more severe, significantly affecting the battery's performance, such as capacity, rate characteristics, and cycle life.

### 4.3 Conductive additive interface failure

During the lithium-insertion process of Si, the active particles squeeze each other due to volume expansion. When Si undergoes lithium-extraction, the volume shrinks, and the internal morphology of the electrode plate is difficult to restore. This easily leads to the separation of the active material and the conductive agent. This continuous volume change makes the contact at the interface unstable, gradually damaging the originally effective conductive channels.^65^ As the number of cycles increases, the bond between silicon and the conductive additive becomes less tight, and the resistance at the interface increases sharply. This failure phenomenon seriously hinders the transmission of electrons, significantly increasing the internal resistance of the battery, greatly reducing the charge–discharge efficiency, and also affecting the rate performance of the battery. In addition, interface failure increases the non-uniformity of the electrode structure. In some areas, silicon cannot fully participate in the electrochemical reaction, thus accelerating the attenuation of the battery capacity and shortening the cycle life of the battery.

## 5 Improvement measures for silicon-based anode materials

Silicon-based anode materials have become one of the current research hotspots of anode materials for lithium-ion batteries due to their relatively high theoretical specific capacity. However, they undergo severe volume expansion during the charging and discharging process, leading to problems such as capacity fading, which restricts their further development in the battery industry. To improve the electrochemical performance of silicon, researchers have adopted a variety of modification methods. The following are some common directions of modification research.

### 5.1 Nanostructuring

#### 5.1.1 0D silicon nanoparticles (SiNPs)

**Fig. 5:**
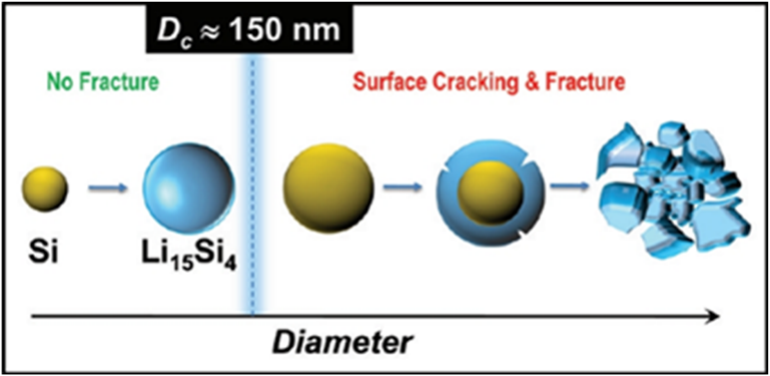
Schematic diagram of the critical fracture size of silicon nanoparticles.^66^

0D silicon nanoparticles have extremely small sizes, with dimensions in all directions in three-dimensional space being very small, approaching a point-like shape, similar to a point-particle. They are typically in the range of a few to several tens of nanometers. Their shapes depend on the synthesis methods and conditions, and can be spherical, cubic, or other irregular shapes. The crystal structures include single-crystal, polycrystalline, or amorphous states. Due to a large number of atoms located on the surface, their surface energy increases,^67^ endowing them with high reactivity. Moreover, the surface charge distribution has a significant impact on the dispersion of the particles and their interaction with other substances. In the research of lithium-ion batteries, it has been found that when silicon undergoes volume expansion, it is not uniform in all directions, but mainly concentrated in the <110> direction. During lithium insertion and extraction, Lee *et al.*^68^ found that the expansion of crystalline silicon nanocolumns in the <110> direction is significantly higher than that in other directions such as <100>. Further experiments show that even at a relatively low lithiation rate of 0.1 mV s^−1^, particles with a diameter greater than 360 nm are prone to cracking due to the volume effect, while particles with a diameter less than 240 nm hardly crack at the same lithiation rate. At a relatively high lithiation rate of mV s^−1^, their degree of cracking is also much lower than that of the 360 nm samples. The research by Liu *et al.*^66^ indicates that nanostructuring enables rapid lithium transport and rapid stress relaxation. When the size of the silicon material is less than 150 nm, the internal stress generated by the volume expansion of silicon due to ion insertion is insufficient to drive the further propagation of cracks (Fig. 5). At this time, the silicon material will not break or pulverize. Therefore, for silicon materials applied to silicon-based anodes, at least one dimension should be less than 150 nm. Silicon-based anode materials with this as the matrix can effectively address the volume expansion of silicon during the lithium-insertion and extraction processes and have an ideal lithium-insertion capacity.^69^ These research results provide important guiding directions for improving the performance of silicon-based anode materials in lithium-ion batteries. By deeply understanding the anisotropy of silicon volume expansion and the nanoscale effect, it is expected to develop more efficient and stable lithium-ion batteries in the future, promoting the development of related fields.

#### 5.1.2 1D silicon nanowires (SiNWs) and silicon nanotubes (SiNTs)

**Fig. 6:**
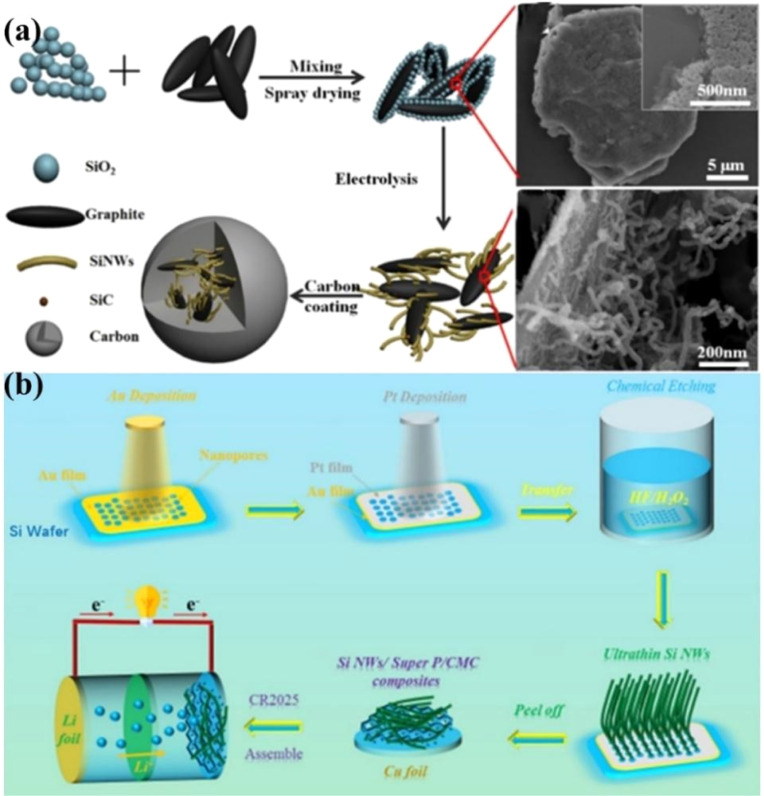
(a) The preparation process of SiNWs/graphite composite materials and the SEM images of the mixed powder before and after electrolysis.^70^ (b) The process of synthesizing UTSiNWs by the BACE method.^71^

Compared with 0D silicon nanoparticles, silicon nanowires have the advantages of easy stress dispersion and high-efficiency electron transfer. Keller and his colleague^72^ prepared silicon nanoparticles (SiNP) and silicon nanowires (SiNW) of varying sizes by means of laser pyrolysis and gold-catalyzed vapor–liquid–solid (VLS) growth methods respectively, with a focus on the application of silicon as an anode material for lithium batteries. The research reveals that the size and shape of SiNP and SiNW exert a significant influence on their electrochemical performance. SiNP boasts a relatively high specific capacity, yet its cycling stability is poor. The initial coulombic efficiency exhibits a linear correlation with the specific surface area. Large-sized SiNP tend to form the crystalline phase Li_15_Si_4_ and undergo electrochemical sintering, which results in capacity degradation. Although SiNW has a lower specific capacity, the SiNW9 electrode with the smallest size features a high coulombic efficiency and excellent capacity retention. The one-dimensional (1D) structure offers advantages in terms of maintaining the three-dimensional (3D) porous structure of the material, facilitating lithium diffusion, and preventing electrochemical sintering. SiNWs can grow on metal substrates and be directly connected to the current collector without additional binders or conductive additives.^73,74^ They also provide a direct 1D electron path for rapid charge transfer and can accommodate large volume changes, preventing material fragmentation.^75,76^ Similar to other nanostructures, the drawback of Si-NWs is related to their high surface-to-volume ratio, which leads to a high irreversible capacity. Prosini *et al.*^77^ successfully synthesized silicon nanowires (SiNWs) with diameters in the range of 200 to 500 nanometers through the chemical vapor deposition (CVD) method using gold (Au) as a catalyst and used them as anode materials for lithium-ion batteries. The experimental results show that at a low current density of 0.05 mA, the initial discharge capacity of the Si-NWs anode material is approximately 2.15 mA h, and it shows a good capacity retention rate in subsequent cycles. Although an irreversible capacity of 0.3 mA h was observed in the first cycle, as the charging current increases, the material exhibits a lower capacity decay. In addition, a lithium-ion battery constructed by pairing Si-NWs with a LiFePO_4_ cathode material was tested at a current density of 0.12 mA. The initial discharge capacity was 0.8 mA h, and after 10 cycles, the capacity slowly decreased to 0.6 mA h, with the coulombic efficiency increasing to 95%. The capacity decay of the battery is considered to be related to poor battery matching, resulting in the consumption rate of lithium ions in the cathode material exceeding their re-insertion rate in the anode. Yu *et al.*^70^*in situ* grew silicon nanowires (SiNWs) on the surface of graphite by molten-salt electrolysis and prepared a silicon nanowire/graphite composite material (SiNWs/G@C) for use as an anode material for high-performance lithium-ion batteries (Fig. 6a). It was found that at a current density of 0.5C, the SiNWs/G@C anode material exhibited an initial discharge capacity of approximately 674.4 mA h g^−1^ and a capacity retention rate of up to 90.04% after 100 cycles. This excellent performance is attributed to the formation of silicon carbide (SiC) connections between the silicon nanowires and graphite in the composite material. These connections improve the mechanical strength and cycling stability of the material. By adjusting the charge ratio during the electrolysis process, the content of SiC can be controlled, thereby optimizing the performance of the material. Sun *et al.*^71^ produced ultra-thin silicon nanowires (UTSiNWs) with an average diameter of 30 nm and 10 nm through a bimetal-assisted chemical etching method and used them as anode materials for lithium-ion batteries (Fig. 6b). It was found that the 30 nm UTSiNWs showed more excellent stability and capacity retention rate than 100 nm silicon nanowires during cycling. Specifically, the 30 nm UTSiNWs had a discharge capacity of 1066.0 mA h g^−1^ at a current density of 300 mA g^−1^, and the capacity retention rate was as high as 87.5% after 50 cycles. This excellent performance is attributed to its smaller average diameter and more effective SiO_*x*_ content, which helps to form a stable solid-electrolyte-interface (SEI) layer during cycling and reduce interfacial reactions.

**Fig. 7:**
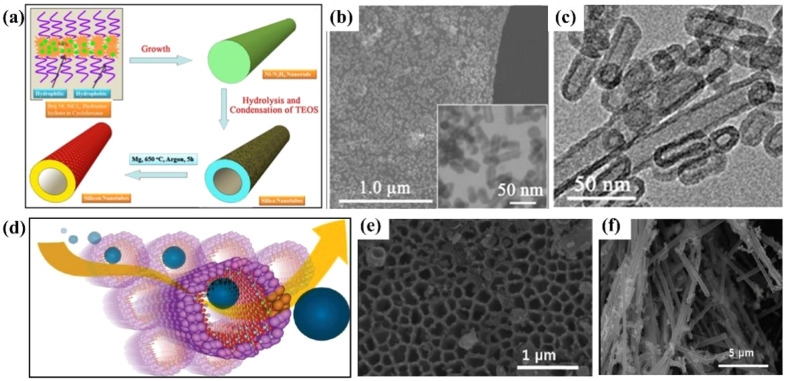
(a) Schematic diagram of the synthesis of silicon nanotubes. (b) SEM image and TEM image (inset) of silicon nanotubes. (c) SEM image of silicon nanotubes;^78^ (d) schematic diagram of the lithium-ion path in silicon nanotubes. (e) Top-view FE-SEM image of silicon nanotubes. (f) SEM image of cycled silicon nanotubes (silicon nanotubes extracted from a lithium-ion battery after 200 cycles).^79^

Silicon nanotubes are hollow tubular structures composed of silicon atoms, featuring a unique hollow tubular morphology and a nanoscale tube diameter. Silicon nanotubes can effectively buffer the volume change of silicon during the charging and discharging process. Their hollow structure provides space for the expansion of silicon, reducing the risk of structural damage caused by the volume effect.^80^ Compared with other materials, silicon nanotubes have a larger specific surface area, enabling them to fully contact the electrolyte and providing more active sites for electrochemical reactions. Lithium ions can be relatively easily inserted into the surface of silicon nanotubes, and due to their nanoscale tube diameter, the diffusion distance of lithium ions is shorter, resulting in relatively less polarization and capacity decay of silicon at high rates. At the same time, silicon nanotubes can promote the penetration of the electrolyte, providing a smoother channel for charge transfer and improving the charge–discharge efficiency of the battery. However, the preparation of silicon nanotubes is difficult, usually requiring complex processes and expensive equipment, resulting in a high cost. Silicon nanotubes can accommodate large volume changes related to lithium and achieve reversible behavior during morphological changes, thereby improving the cycle retention rate and reliable operation of the battery. Wen *et al.*^78^ prepared silicon nanotubes by first preparing silica nanotubes using rod-like as Ni–N_2_H_4_ a template and then converting them into silicon nanotubes *via* a magnesiothermic reduction process assisted with magnesium powder, and used them as anodes for lithium-ion batteries (Fig. 7a). They found that these silicon nanotubes had better electrochemical performance compared to commercial silicon meshes. The silicon nanotubes prepared by a specific method have a one-dimensional structure, with a diameter of approximately 15 nm, a length of 50–200 nm, a good crystal structure, and certain pore characteristics (Fig. 7b and c). At 0.5C, the capacity loss of the silicon nanotube electrode was less than that of the Si-325 mesh electrode. Initially, the capacities of both decreased due to the formation of the solid electrolyte interface (SEI), irreversible lithium insertion/extraction, electrode pulverization, and defect structures in the silicon material, with the capacity decline of the Si-325 silicon mesh being more significant. After the 6th cycle, the performance of the silicon nanotubes became relatively stable, and the discharge capacity in the 10th cycle was approximately twice that of the Si-325 silicon mesh. In terms of rate performance, the capacity retention rate of silicon nanotubes at different rates was higher than that of the Si-325 mesh, and they still had a relatively high capacity after 90 cycles. Although their capacity was lower than that reported in some cases, possibly due to incomplete reduction of silica nanotubes, the nanotube structure and the amorphous matrix gave them advantages in alleviating volume changes, increasing electrolyte contact, and stabilizing the interface, making their performance superior to that of commercial silicon meshes and graphite electrodes. Park *et al.*^79^ prepared silicon nanotubes for use as anodes in lithium-ion batteries through carbon-coating deposition (Fig. 7d–f). In a half-cell, at a rate of 0.2C, the first-discharge capacity reached 3648 mA h g^−1^, and the charge capacity was 3247 mA h g^−1^, demonstrating a coulombic efficiency as high as 89%. When the charging rate was increased to 5C (*i.e.*, 15 A g^−1^), the charge capacity could still reach 2878 mA h g^−1^. A full-cell was constructed with LiCoO_2_ as the cathode and silicon nanotubes as the anode, and the initial capacity was higher than 3000 mA h g^−1^ at both 3C and 5C rates. Notably, after 200 cycles at a 1C rate, the capacity retention rate was as high as 89%, showing significant advantages compared with commercial graphite batteries and indicating its high performance and stability in long-term cyclic use. The high capacity is attributed to the large active surface area provided by the porous SiNTs array structure, which is conducive to tolerating large volume expansions during the alloying/de-alloying reactions, thus promoting the storage of Li^+^ according to the pseudocapacitance mechanism.

#### 5.1.3 2D silicon nanofilm and silicon nanosheet

**Fig. 8:**
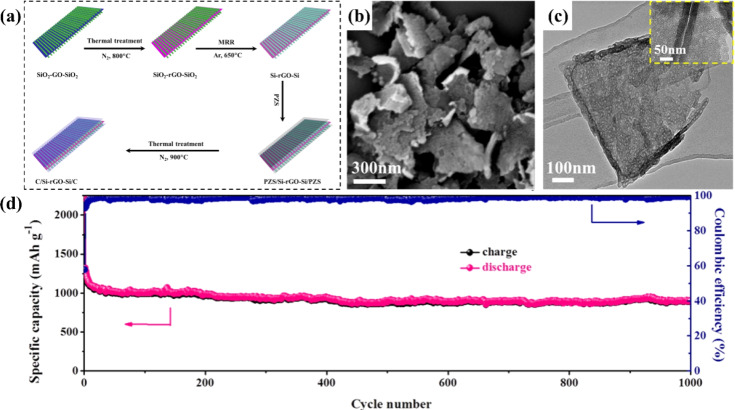
(a) For 2D sandwich-like C/Si-rGO-Si/C nanosheets: (a) schematic diagram of the manufacturing process (b) SEM (c) TEM (d) long-term cycling performance diagram of 1000 cycles at 1 A g^−1^.^81^

2D silicon nanofilms have a nanoscale thickness, typically ranging from a few nanometers to several tens of nanometers. They are in a two-dimensional planar shape, with atoms or molecules arranged within the plane. There are mainly two preparation methods for 2D silicon nanofilms. One is chemical vapor deposition (CVD), in which gaseous precursors decompose under the action of high temperature and a catalyst, and silicon atoms deposit on the substrate surface to form a film.^82^ The other is physical vapor deposition (PVD), in which the silicon source material is vaporized through physical processes such as evaporation and sputtering, and then deposited on the substrate.^83–85^ Suresh *et al.*^86^ used the a methanol-mediated floating catalyst chemical vapor deposition (FCCVD) method to fix a silicon film on a carbon nanotube macroscopic film (CNM) current collector, and then covered the film with a single layer of graphene. This delamination-inhibited Si film with a graphene capping layer had a long cycle life (>1000 charge–discharge steps) and an average specific capacity of 806 mA h g^−1^. The average capacity after 1000 charge/discharge cycles could still remain at 2821 mA h cm^−3^. Silicon nanosheets are another two-dimensional silicon anode structure. Compared with other shaped silicon nanomaterials, their advantage is that Li ions have a shorter transport path perpendicular to their plane direction, which is more conducive to ion diffusion and charge transfer.^87^ Compared with silicon nanofilms, silicon sheet anodes have a wider variety of process routes available in the preparation method. Common methods include etching and exfoliation, chemical vapor deposition, and templating. Yao *et al.*^81^ successfully fabricated a novel two-dimensional porous sandwich-like silicon/carbon nanosheet, in which a porous silicon nanomembrane grew on both sides of reduced graphene oxide (rGO), and then a carbon layer was coated (denoted as C/Si-rGO-Si/C)(Fig. 8a–c). The co-existence of micropores and mesopores in the C/Si-rGO-Si/C nanosheets provided conditions for the rapid diffusion of Li^+^, and the porous Si provided a short channel for electrical transmission. At the same time, the coated carbon layer could not only promote the formation of a stable SEI layer but also improve the electrical conductivity of the coupling between nanoscale Si and reduced graphene oxide. Therefore, the unique nanostructure endows the obtained C/Si-rGO-Si/C electrode with a high reversible capacity (1187 mA h g^−1^ after 200 cycles at 0.2 A g^−1^), excellent cycle stability (894 mA h g^−1^ after 1000 cycles at 1 A g^−1^)(Fig. 8d), and high-rate capacity (694 mA h g^−1^ at 5 A g^−1^ and 447 mA h g^−1^ at 10 A g^−1^).

#### 5.1.4 3D porous silicon

**Fig. 9:**
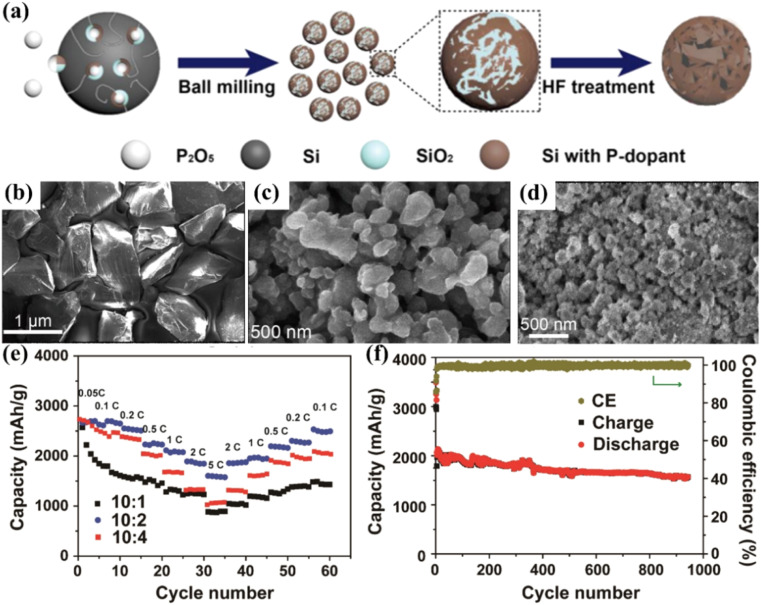
(a) Schematic diagram of the synthesis of porous silicon, which involves two steps: high-energy mechanical milling of Si and P_2_O_5_, and hydrofluoric acid (HF) treatment. (b) SEM image of metallurgical silicon powder (∼98 wt%). (c) SEM image of nano-Si particles with P-dopants and SiO_2_ prepared after ball-milling. (d) SEM image of porous nano-Si particles with P-dopants after acid etching. (e) Cycling performance of porous nano-Si particles with P-dopants prepared from different weight ratios of Si to P_2_O_5_ at different rates ranging from 0.05C to 5C. (f) Cycling performance of the prepared carbon-coated porous nano-Si particles with P-dopants.^88^

Compared with the previous several nanostructures, porous Si has a rich pore structure, which can provide a certain buffer space for the volume change of Si. At the same time, its high specific surface area is also conducive to enhancing the transmission and diffusion of Li^+^, thus promoting the improvement of rate performance and long-cycle performance. Currently, the main preparation methods of porous Si include electrochemical etching,^89–91^ magnesiothermic reduction,^92,93^ templating method,^94,95^*etc.* Lv *et al.*^88^ repared porous nanosilicon particles with controllable dopants by combining ball-milling and acid-etching methods (Fig. 9a–d). Compared with intrinsic silicon, the porosity of the porous nanosilicon is as high as 45.8%, and the electrical conductivity has changed by 9 orders of magnitude. The experimental results show that the prepared nanosilicon particles can maintain 2000 mA h g^−1^ at a current density of 0.5C for 100 cycles, maintain an excellent rate performance of 1600 mA h g^−1^ at a current density of 5C, and maintain a stable rate performance of over 1500 mA h g^−1^ at a current density of 1C for 940 cycles (Fig. 9e and f). Yao *et al.*^96^ synthesized interconnected silicon hollow sub-microspheres through a silicon-templated SiH_4_ deposition and selective etching process, achieving a high initial discharge capacity of 2725 mA h g^−1^. During 700 cycles, the capacity decline per 100 cycles is less than 8%. The silicon hollow-sphere anode also shows a coulombic efficiency of 99.5% in the later cycles. The high-reversible-capacity hollow structure and porous shell can not only relieve the huge volume change but also facilitate the diffusion and transportation of Li^+^ to the active material.

### 5.2 Composite materials1

#### 5.2.1 Silicon–carbon composite (Si–C)

**Fig. 10:**
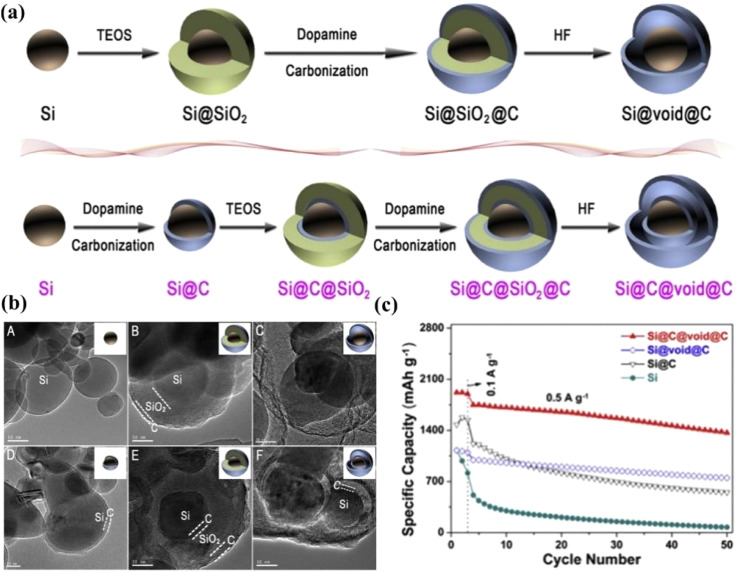
(a) Schematic diagrams of the preparation of traditional yolk–shell Si@void@C and the novel core–shell yolk–shell Si@C@void@C. (b) TEM images of different Si and Si/C materials. (c) Cycling performance diagrams of Si, Si@C, Si@void@C, and Si@C@void@C at an electrode current of 0.5 A g^−1^.^97^

Carbon materials, on the other hand, possess certain flexibility and structural stability. When silicon is combined with carbon, carbon can act as a buffer matrix, alleviating to some extent the stress caused by the volume expansion of silicon, reducing the damage to the electrode structure, and thus improving the cycling stability.^98^ Carbon materials generally have good electrical conductivity, such as graphite, graphene, carbon nanotubes, and amorphous carbon. In contrast, silicon has relatively poor electrical conductivity. Carbon can provide a rapid electron-transfer channel for the composite material, enhancing its overall electrical conductivity, reducing the internal resistance of the battery, and facilitating the improvement of the battery's charge–discharge efficiency and rate performance.^99^ Du *et al.*^100^ obtained a core–shell Si@C nanocomposite by covalently grafting aniline monomers onto the Si surface followed by carbonization. After 100 cycles, the reversible capacity remained above 750 mA h g^−1^, which was superior to the cycling performance of pure silicon. The enhanced performance is attributed to the fact that the uniform and elastic carbon coating can effectively improve the electronic conductivity and adapt to the severe volume changes of Si particles. Xu *et al.*^101^ synthesized watermelon-shaped Si/C microspheres with a hierarchical buffer structure and optimized the size distribution. At 0.1C, the discharge and charge capacities of the Si/C anode were 695 and 620 mA h g^−1^ respectively, with an initial coulombic efficiency of 89.2%. The Si/C anode exhibited excellent cycling stability under high tap density. After 500 cycles, the reversible areal capacity was still higher than 1.91 mA h cm^−2^. It also showed good cycling performance and rate performance at different temperatures (−20, 25, 55 °C). Even at a high current density of 5C, it could still maintain 80% of the original charge capacity. The excellent electrochemical performance of the Si/C microspheres is attributed to the internal pores in the structure, which can provide the space required for the volume expansion of silicon during the charging and discharging process, thereby reducing the stress caused by the volume change, preventing the cracking and pulverization of silicon particles, and maintaining the relative stability of the electrode structure. Xie *et al.*^97^ successfully prepared a core–shell–yolk–shell Si@C@void@C nanocomposite structure (Fig. 10a and b). Compared with Si@void@C, the newly proposed structure introduced core–shell nanoparticles Si@C as the yolk. This additional carbon shell can not only reduce the resistance between the silicon yolk and the hollow carbon shell but also effectively protect the silicon yolk from corrosion by the electrolyte. The results show that the Si@C@void@C electrode exhibits significantly enhanced reversible capacity and cycling stability. The initial reversible capacity at 100 mA h g^−1^ was 1910 mA h g^−1^, with a capacity retention rate of 71%. After 50 cycles at 500 mA h g^−1^, the capacity was 1366 mA h g^−1^(Fig. 10c).

Although remarkable achievements have been made in the research and development of traditional carbon-coated silicon–carbon materials with core–shell structures, yolk–shell structures, *etc.*, the exploration of more advanced silicon–carbon composites is still ongoing. New types of silicon–carbon materials are prepared through chemical vapor deposition (CVD). Resin-based or bio-based materials are used as porous carbon sources, and silanes are used as silicon sources. The deposition is carried out in two steps of adsorption and cracking inside the porous carbon. Finally, carbon-source gases such as acetylene and ethylene are introduced to uniformly coat a carbon layer on the surface of the materials. Sung *et al.*^102^ used ethylene to inhibit the growth of silicon crystals and synthesized sub-nanometer silicon anode materials embedded in a dual matrix of silicon carbide and amorphous carbon through chemical vapor deposition. This material shows excellent electrochemical performance. The C(5)Si-G electrode exhibits good performance at a rate of 0.1C, and the coulombic efficiency reaches 99.96% after 50 cycles. The 110 A h^−1^ full cell prepared with this material has good cycle stability (the capacity retention rate is 91% after 2875 cycles) and a long calendar life (97.6% after 365 days). This unique structure has excellent electrochemical performance. On the one hand, the carbon-based material provides a stable support framework for the sub-nanometer silicon, effectively buffering the volume changes of silicon during the lithiation and delithiation processes. At the same time, the formation of Si–C bonds slows down the growth of the silicon core and inhibits the unfavorable phase evolution, thus significantly improving the cycle stability. Despite the many advantages of CVD-deposited silicon–carbon materials, due to the limitations of technical barriers such as the selection of porous carbon, the cost of silanes, and the deposition process, large-scale and continuous production remains an industrialization challenge for enterprises producing new silicon–carbon materials. It will still take some time to improve the relevant equipment and optimize the process.

#### 5.2.2 Silicon–metal/metal oxide/metal sulfide composite

**Fig. 11:**
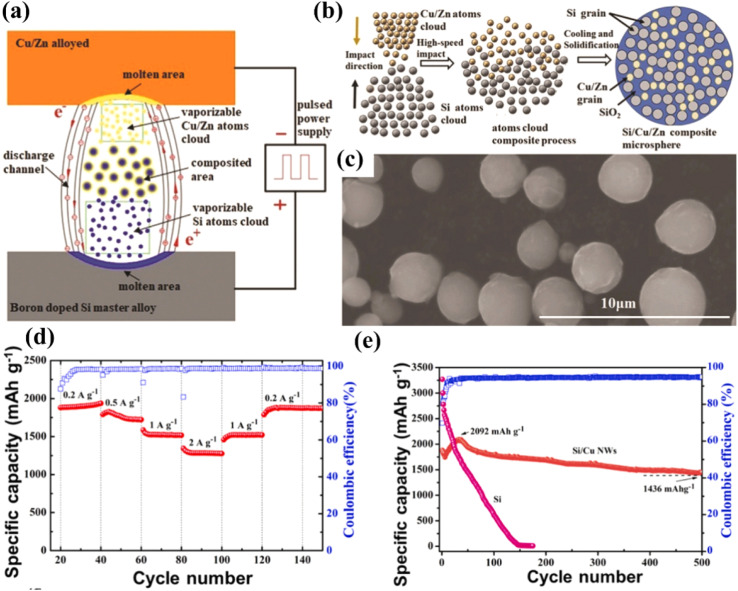
Si/Cu NW: (a) schematic diagram of the pulsed-discharge method, and (b) preparation process of Si/Cu/Zn. (c) SEM image of Si/Cu/Zn ternary microspheres. (d) Rate performance. (e) Cycling performance.^103^

Metals generally have good electrical conductivity. When combined with silicon, they can reduce the internal resistance of the battery and enhance the rate performance. Some malleable metals can also buffer the volume change of silicon to a certain extent, enhance the mechanical strength, and reduce the damage to the electrode structure. In the Si/Cu alloy, the Cu matrix can not only provide excellent electrical properties, but also the high elastic modulus of the Cu matrix is inactive to lithium ions during cycling, which further maintains the structural stability of the Si/Cu electrode.^104^ Hong *et al.*^103^ prepared silicon/copper (Si/Cu) nanowires as high-performance anodes for lithium-ion batteries through a pulsed electric discharge method and chemical etching. Using a P-type silicon master alloy as the positive electrode and a Cu/Zn alloy as the negative electrode, pulsed electric discharge was carried out to form Si/Cu/Zn microspheres. Subsequently, Si/Cu nanowires were obtained through chemical etching and purification (Fig. 11a–c). The prepared Si/Cu nanowires have specific size and structural characteristics, and exhibit excellent performance in electrochemical performance tests. At a current density of 2000 mA h g^−1^, the specific capacity reached a peak of 2092 mA h g^−1^ after 30 cycles, and still remained at 1456 mA h g^−1^ after 500 cycles, with only a 30% capacity decline (Fig. 11d and e). These results indicate that Si/Cu nanowires have great application potential in lithium-ion batteries.

Metal oxides (such as titanium dioxide, iron oxide, *etc.*) can act as buffer layers to alleviate the volume expansion of silicon. They have certain structural stability and can accommodate part of the volume change of silicon. Some metal oxides can also react with the electrolyte to form a stable solid-electrolyte-interface film, reducing the decomposition of the electrolyte and side reactions, and improving the cycling performance of the battery. Lotfabad *et al.*^105^ coated the surface of silicon nanowires (SiNWs) with TiO_2_ by atomic layer deposition (ALD) to prepare a TiO_2_/SiNW nanocomposite as the anode of a lithium-ion battery. In this material, by changing the thickness of the TiO_2_ coating, it has different structures, which can improve the cycling stability and coulombic efficiency of the composite. At a rate of 0.1C, the capacity retention rate of (10)TiO_2_-200/SiNWs after 100 cycles reached 58%, which is higher than 30% of bare SiNWs. At 5C, (10)TiO_2_-200/SiNWs could maintain 34% of the initial capacity. This is attributed to the special structure formed by the TiO_2_ coating and its influence on the SEI layer, making the composite material perform excellently in terms of electrochemical performance.

Metal sulfides (such as molybdenum disulfide, tungsten disulfide, *etc.*) have a relatively high theoretical specific capacity. Their synergistic effect with silicon can increase the specific capacity of the composite material. The layered structure of some metal sulfides is conducive to the insertion and extraction of lithium ions, which can improve the rate performance of the electrode. At the same time, it can also buffer the volume change of silicon to a certain extent and enhance the cycling stability of the electrode.^106^ Molybdenum disulfide (MoS_2_) is a classic transition-metal sulfide. Its S–Mo–S interlayer structure is connected by weak van der Waals forces. Its graphite-like layered distribution allows for more Li-ion insertion and extraction. During the lithiation/delithiation process, the volume expansion rate of MoS_2_-based materials is only 103%. Moreover, the smaller the volume expansion rate, the better the cycling performance and lithium-storage capacity. Marriam *et al.*^107^ synthesized two-dimensional MoS_2_ nanosheets with a heterostructure on a water-soluble NaCl substrate. These nanosheets can not only adapt to the volume expansion of silicon particles but also possess excellent electrochemical properties. The long-term cycling stability of the heterostructured MoS_2_@Si electrode is much better than that of MoS_2_ and Si. In particular, in the 500th cycle, MoS_2_@Si exhibited excellent cycling stability. At a high current density of 500 mA g^−1^, its capacity retention rate was 60%, while that of MoS_2_ was 21% and that of pristine Si was 0.3%. Compared with pristine Si (about 430%), a significant reduction in volume expansion (68%) was observed in the MoS_2_@Si anode. The method for synthesizing two-dimensional MoS_2_ nanosheets and their application in the sandwich-like heterostructure add advantages to the application of MoS_2_ in energy storage. Kawade *et al.*^108^ successfully synthesized a flexible graphene-like layered MoS_2_-G structure as an ideal substrate for silicon nanoparticles. The MoS_2_-G nanoparticles sandwiched between the layers have sufficient space, which facilitates the transmission of lithium ions and also restricts the volume expansion during the lithiation and attenuation processes. The Si/MoS_2_ nanocomposite has a reversible discharge capacity of 1549 mA h g^−1^ in the voltage range of 0.01–3.0 V, which is higher than that of MoS_2_-G, and has better stability than pristine silicon. At the same time, the Si/MoS_2_ nanocomposite provided the highest and most stable reversible capacity of 923 mA h g^−1^ in 90 cycles at 200 mA g^−1^. More importantly, by integrating the advantages of nanostructure engineering and hybridization, the diffusion path length was shortened, better lithium-diffusion kinetics were observed, and ultimately, the reversible capacity and cycling performance were improved.

#### 5.2.3 Silicon-conductive polymer composite

**Fig. 12:**
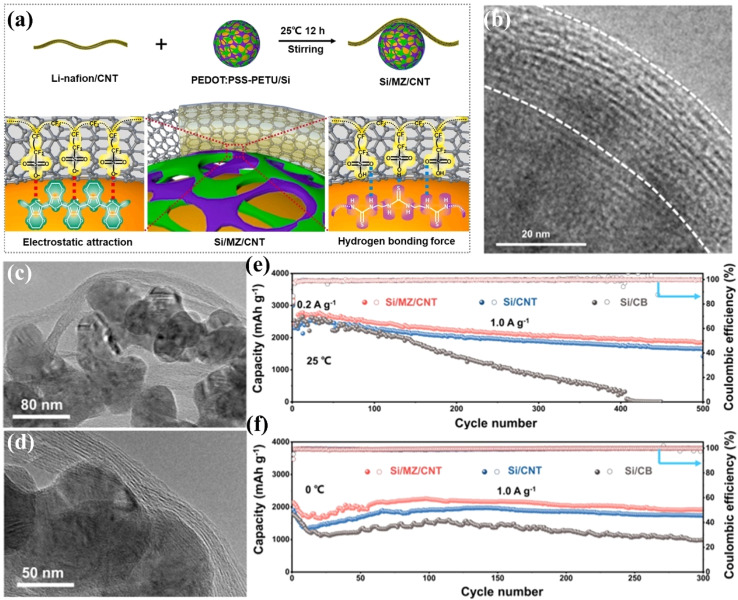
(a) Schematic diagram of the dynamically stable interface by the molecular-zipper fixation strategy. (b) Enlarged TEM image of the dynamically interface-modified Si/MZ/CNT. (c and d) TEM images. Galvanic charge curves of Si/MZ/CNT, Si/CNT and Si/CB anodes at a current density of 1.0 A g^−1^ at (e) 25 °C and (f) 0 °C.^109^

Non-conductive binders inevitably reduce the electrical conductivity and/or ionic conductivity of the anode to some extent. Conductive polymers have good electrical conductivity, flexibility, and processability. They can alleviate the volume change problem of silicon to a certain extent and improve the electrical conductivity and stability of the electrode.^110,111^ Su *et al.*^109^ constructed a dynamically stable interface between nanosilicon and carbon nanotubes (CNTs) through a molecular zipper fastening strategy. This interface was formed by cross-linking a binary polymer (PEDOT: PSS-PETU) and lithiated perfluorosulfonic acid (Li-nafion), enabling the CNTs to be tightly locked on the surface of nanosilicon (Fig. 12a–d). The silicon anode with a dynamically stable interface (Si/MZ/CNT) exhibited high capacity, high-rate performance, and long-cycle stability at both room temperature and freezing temperature. The capacity remained at 1850 mA h g^−1^ after 500 cycles at 25 °C, and was 1917 mA h g^−1^ after 300 cycles at 0 °C (Fig. 12e–f). In addition, this material had a high lithium-ion diffusion coefficient, low charge-transfer impedance, and a stable SEI layer. Compared with traditional Si/CNT and Si/CB, Si/MZ/CNT had better structural stability and mechanical properties. This study provided an effective strategy for improving the performance of silicon-based anode materials. Fan *et al.*^112^ prepared an integrated anode composed of anode material particles and a all-carbon binder reinforced by CNTs through a slurry-casting and heat-treatment method. The all-carbon binder, including PVDF-derived carbon and CNTs, had a higher binding ability compared with the traditional PVDF binder. It could effectively suppress the volume expansion of the anode material and maintain the stability of the electrode structure. The integrated electrode with SnO_2_ as the active material (SnO_2_–C-CNT) exhibited excellent electrochemical performance. After 500 cycles, it could still maintain a high capacity of 861.4 mA h g^−1^ at 0.5C. In addition, this all-carbon binder strategy could effectively improve the cycling stability of SnO_2_ with different sizes and morphologies, as well as silicon-based electrodes. This study provided a new and effective way to solve the problem of anode materials with large volume expansion. Xiao *et al.*^113^ designed a multifunctional cross-linked composite binder (LPTS) for silicon-based anodes. LPTS was composed of lithiated polyacrylic acid (LiPAA), sericin (SS), and tannic acid (TA). A strong three-dimensional network was formed through multiple hydrogen bonds and covalent cross-linkages, which could effectively relieve the volume stress of silicon during cycling. The-COOLi groups in LiPAA could provide an additional source of lithium ions and fast ion-diffusion channels, enhancing the ionic conductivity of the electrode. The multiple functional groups of SS formed hydrogen bonds with LiPAA, and TA enhanced the degree of cross-linking and improved the interfacial compatibility with carbon. The LPTS binder enabled the silicon electrode to have a high initial coulombic efficiency, excellent rate performance, and good cycling stability. It was also applicable to silicon–carbon anodes, ensuring the stable cycling of the electrode at a high areal capacity. After 70 cycles at 0.2C, the capacity retention rate was 97.3%. After 200 cycles at 0.5C, the capacity retention rate of the Si–C/LPTS full-battery was 72.2%. This study provided new ideas for the development of efficient and low-cost silicon-based binders and had important application potential for high-energy-density systems.

### 5.3 Development of silicon-containing precursors

#### 5.3.1 Naturally-occurring substances containing SiO_2_

Many natural minerals contain silica, such as quartzand^114,115^ diatomaceous earth.^116,117^ These minerals are abundant in nature and easily accessible, which makes the cost of raw materials relatively low and helps to reduce the overall production cost of lithium-battery materials. At the same time, natural SiO_2_ has a stable chemical structure. During the charging and discharging process of the battery and under different environmental conditions, it can maintain good stability, is not prone to decomposition or other chemical reactions, and can ensure the stable performance and safety of the battery. Therefore, it is of great significance for the research on silicon-containing substances and the development of new energy materials.

#### 5.3.2 Organosilicon compounds

Organosilicon compounds refer to compounds that contain a carbon–silicon bond (Si–C) in the molecule, with at least one organic group directly connected to the silicon atom. Sometimes, compounds in which organic groups are indirectly connected to the silicon atom through oxygen, sulfur, nitrogen, *etc.* are also regarded as organosilicon compounds. Their electrochemical properties can be regulated by changing their chemical structures and functional groups.^118,119^ Compared with other silicon sources, organosilicon compounds can more easily yield SiO_*x*_C derivatives with uniform composition, structure, and morphology, which helps to achieve precise control over high-energy-density silicon-based anode materials during the preparation process. A common synthesis strategy is to combine organosilicon compounds with a sol–gel method and a carbonization reaction, thereby converting them into SiO_*x*_C derivatives.

### 5.4 The prelithiation technology of silicon-based anode materials

**Fig. 13:**
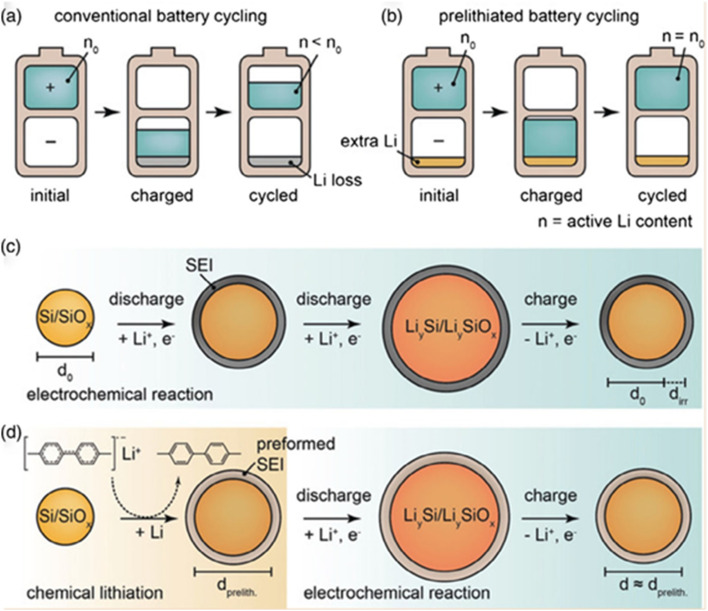
Schematic diagram of the principle of pre-lithiation technology. (a) In the initial electrochemical cycles of the anode of a traditional lithium-ion battery with a low ICE, there is a permanent loss of active lithium. This leads to a net energy loss due to the insufficient utilization of the cathode capacity. (b) Achieve the maximum energy density of the full battery through direct contact prelithiation to obtain the ideal ICE. (c) Capture the irreversible lithium through electrochemical prelithiation, which promotes the formation of the SEI. (d) Conduct lithiation/delithiation through chemical prelithiation. Volume expansion and the loss of active lithium occur before the battery is assembled.^120^

Nanostructuring silicon materials or preparing porous materials can effectively increase their specific surface area, expand the contact interface with the electrolyte, enhance the reaction activity, shorten the lithium-ion transport distance, and facilitate lithium-ion diffusion. However, a large specific surface area will increase the interface for side reactions. In particular, it is prone to forming more solid-electrolyte-interface (SEI) films, causing problems such as low initial coulombic efficiency and poor cycling stability in practical applications. As shown in the Fig. 13a–d, prelithiation technology, as one of the effective ways to solve these problems, can compensate for the irreversible capacity loss by introducing lithium into the silicon-based anode material in advance, either before battery assembly or during the initial charging process. This improves the initial coulombic efficiency and cycling stability. There are mainly three methods of prelithiation technology for anodes: chemical prelithiation, electrochemical prelithiation, and mechanical prelithiation.

#### 5.4.1 Direct contact prelithiation

Direct contact prelithiation technology has strong applicability and can be used for prelithiation of other types of electrodes (carbon-based). Direct contact prelithiation mainly uses lithium metal powder and Li_*x*_Si materials as lithium sources, which are deposited on the silicon anode through direct contact.^121,122^. Due to the potential difference formed between the silicon anode and the positive electrode of the prelithiation reagent, an electric field will be generated. Under the action of the electric field, electrons will move from the low-potential region to the high-potential region at the contact points. In order to maintain electrical neutrality, the prelithiation reagent will release lithium ions, and the lithium ions are embedded into the anode material through the electrolyte to complete the lithiation process. An appropriate amount of lithium diffuses into the silicon, ensuring the formation of a solid electrolyte interface (SEI) film on the surface of the silicon anode. Meng *et al.*^123^ placed a flexible resistive buffer layer (RBL) with good electrical conductivity between the SiO_*x*_ anode and the lithium foil. The RBL consists of a carbon nanotube film coated with polyvinyl butyral (PVB). During direct contact prelithiation, due to the roughness of the Li metal and the electrode surface, electron transfer occurs and diffuses only at the contact points, and the strong reducing property of lithium metal also causes non-uniform prelithiation in the vertical direction. The RBL has a porous structure, high electrical conductivity, and soft characteristics, which can make the lithium foil and the anode come into close contact, achieving uniform and efficient prelithiation of the anode. As shown in the figure, in this RBL layer, with the modification of RBL, the distribution of Li and C is more uniform, indicating more uniform prelithiation. A stable SEI is formed, and there are no micro-cracks and broken powder on the surface of the silicon-based material. After prelithiation, the coulombic efficiency of the full battery increases from 68.9% to 87.3%. After 200 cycles, the capacity retention rate of the prelithiated NCM622‖SiO_*x*_ full battery is 74%, with a capacity of 128 mA h g^−1^, which is much better than the 66 mA h g^−1^ of the non-prelithiated full battery. Watanabe *et al.*^124^ improved the direct contact method by using a laminated battery composed of through-hole cathodes and anodes with diameters of 20, 100, and 200 mm respectively. They found that by optimizing the prelithiation charge amount, capacity balancing time, and pore diameter, the loss of active lithium can be significantly reduced. However, lithium metal powder and Li_*x*_Si materials are quite chemically reactive, and they need to be handled in an environment free of water and oxygen. Meanwhile, the uneven distribution of contact points between the prelithiation reagent and the electrode often leads to uneven prelithiation. The lithiated silicon-based electrode is also extremely sensitive to environmental humidity, and improper subsequent operations may trigger serious safety accidents. From a safety perspective, applying direct contact prelithiation to industrial battery production will significantly increase the safety cost.

#### 5.4.2 Electrochemical prelithiation

Electrochemical prelithiation is a prelithiation method that transfers lithium from a lithium source (such as a lithium metal electrode) to the electrode material through electrochemical reactions in the battery. During the electrochemical prelithiation process, the electrode to be prelithiated is usually combined with a lithium source electrode to form an electrochemical cell. Charging and discharging are carried out under certain current density, voltage and time conditions, enabling lithium to be embedded from the lithium source electrode into the electrode material to be prelithiated. At the laboratory level, electrochemical prelithiation is an effective prelithiation technique. It can control the degree of prelithiation relatively precisely and adjust the amount of prelithiation as needed. Su^125^ and his colleagues verified the feasibility of this prelithiation technology. Specifically, before assembling the NCM811‖Si@N-ECGB full cell, the Si@N-ECGB anode was preliminarily lithiated through electrochemical deposition technology in a half cell. Thanks to the increase in lithium storage (20% of the capacity), the NCM811‖Si@N-ECGB full cell has a reversible capacity as high as 170 mA h g^−1^, and the capacity retention rate is 84% after 100 cycles. This work provides the possibility for improving the performance of full cells with silicon-based materials at the laboratory level. However, in the industrialization process, prior to the normal charging and discharging process, electrochemical prelithiation requires additional procedures, including the disassembly and subsequent reassembly of the battery. At the same time, the increase in lithium storage capacity through electrochemical prelithiation is very limited and cannot meet the actual application requirements of industrial production.

#### 5.4.3 Chemical prelithiation

In order to develop mild prelithiation reagents and reduce operational complexity and safety risks, chemical prelithiation has received much attention due to its higher operational safety. Chemical prelithiation is a technology that pre-embeds lithium into silicon-based anode materials by using chemical reagents to chemically react with them, so as to compensate for the irreversible capacity loss of silicon during the first charge–discharge process and improve the initial coulombic efficiency and cycling stability of the battery. Commonly used chemical reagents include lithium metal, lithium compounds (such as LiH, Li_2_O, *etc.*) or organolithium reagents (such as *n*-butyllithium, lithium diisopropylamide, *etc.*). Chemical prelithiation is relatively simple to operate and can achieve a high degree of prelithiation in a relatively short period of time. Shen *et al.*^126^ proposed an effective strategy for chemical prelithiation using lithium naphthalene. By prelithiating S-PAN into a Li_2_S-PAN cathode and prelithiating a silicon nanoparticle anode, a silicon/sulfur lithium-ion battery was successfully constructed. This battery has a high specific energy (710 W h kg^−1^), a high initial coulombic efficiency (93.5%), and good cycling stability. As a prelithiation reagent, lithium naphthalene has advantages such as simple synthesis, mild reaction, and strong prelithiation ability, and can completely lithiate elemental sulfur into Li_2_S. This research provides a new approach for the development of low-cost, environmentally friendly, and high-capacity lithium-ion batteries, and this chemical prelithiation strategy has broad application prospects.

### 5.5 Subsection

In the research of silicon-based anode materials, the four major strategies, namely nanostructuring, composite materials, development of silicon-containing precursors, and prelithiation technology, mainly target issues such as severe volume expansion, poor electrical conductivity, low initial coulombic efficiency, and an unstable solid–electrolyte interface of silicon during the charging and discharging processes. Table 1 shows different structures and types of silicon-based anode materials reported in the literature, classified by modification strategies. Nanostructuring alleviates volume expansion and enhances electrochemical performance by reducing the size of silicon materials and designing special structures. However, it has high preparation costs and complex processes. The composite material strategy involves combining silicon with carbon, metals/metal, oxides/metal sulfides, and conductive polymers, which significantly buffers volume expansion, enhances electrical conductivity, and improves stability. Nevertheless, it has problems such as high-temperature side reactions, reduced electrical conductivity, and high costs. The strategy of developing silicon-containing precursors utilizes natural silicon-containing minerals and organosilicon compounds. The former has a low cost, while the latter is beneficial for precisely controlling material properties. However, it faces challenges in improving the purity of natural minerals and controlling the cost of organosilicon compounds. Prelithiation technology can compensate for irreversible capacity loss and increase the initial coulombic efficiency. However, direct-contact prelithiation has issues such as high reactivity of the lithium source and uneven prelithiation; electrochemical prelithiation has a complex process and limited increase in lithium storage capacity in industrial applications; and some reagents used in chemical prelithiation pose safety risks. Future research needs to focus on solving the existing problems of these strategies, strengthen collaborative innovation among multiple strategies, comprehensively consider material performance, cost, safety, and preparation processes, and contribute to the development of energy storage technology.

**Table 1:** Comparison of the electrochemical performance of silicon-based anodes with different modification strategies in recent publications

Electrode	Modification strategies	Sample	ICE value (%); reversible capacity (mA h g^−1^)	Capacity retention (%) (cycles; rate)	Ref.
0D Si	Nanostructuring	Si@SiO_2_	63; 1215.2	99.4 (50; 0.6 A g^−1^)	127
0D Si		Si/C-CNFs	53.4; 1020.7	51.2 (100; 0.2 A g^−1^)	128
1D Si		30 nm UTSiNWs	—; 933.1	87.5 (50; 0.3 A g^−1^)	129
1D Si		SiNWs/G@C	83.37; 608.5	90.04 (100; 0.5C)	70
1D Si		Sealed Si nanotubes	90; 2169	81 (50; 2C)	130
1D Si		Si nanotubes	89; 3247	89 (200; 1C)	131
2D Si		Si-NSs@rGO	99.5; 1006.1	84.2 (1000; 2 A g^−1^)	132
2D Si		sC/Si-rGO-Si/C	—; 894	70.3 (1000; 1 A g^−1^)	81
3D Si		EPD-5s	97; 1913	73 (100; 0.1C)	133
3D Si		pSi@NC	71.4; 1300	— (200; 1 A g^−1^)	134
Si/C	Composite materials	R-Si/C	—; 501.4	90 (150; 0.5C)	135
Si/C		C@Si/C	46.5; 1233	25.3 (100; 1 A g^−1^)	136
Si/C		NPC/C@Si	—; 592	55 (450; 1C)	137
Si/C		r-Si/4 + 2	—; 1614	30 (300; 0.5C)	138
CVD Si/C		Si-VACNF	76; 1050	30.4 (100; C/1.5)	139
CVD Si/C		SGC	92; 517	96 (100; 0.5C)	140
Si/O		(10)TiO_2_-200/SiNWs	—; 1600	58 (100; 0.1C)	105
Si/Cu		Si/Cu NWs	1456; 86.5	70 (500; 0.2 A g^−1^)	103
Si/S		MoS2@Si	92.3; –	86 (100; 0.1C)	107
SiO_*x*_	Prelithiation technology	PB-SiO_*x*_-60	93.5; 1300	97.7 (250; 1C)	141
SiO_*x*_		NCM622-SiO_*x*_	89.2; 833	75.4 (300; 0.5C)	123

## 6 Summary and prospects

Silicon-based anode materials for lithium-ion batteries have advantages such as high theoretical specific capacity, low lithium-insertion/extraction potential, and excellent fast-charging performance, which have attracted many researchers at home and abroad. An ideal silicon-based anode material should possess the following characteristics: (1) good structural stability: the ideal material can effectively buffer the volume expansion of silicon and prevent the electrode structure from being damaged due to volume effects. (2) Interface stability: a stable, uniform, and thin solid-electrolyte-interface (SEI) film can form on the surface of the ideal material. The stable SEI film can prevent further decomposition of the electrolyte and reduce the consumption of lithium ions. (3) High electrical conductivity: the ideal silicon-based anode material should have high electrical conductivity. By compounding with other conductive materials, an efficient electron-transfer channel can be constructed. (4) High initial coulombic efficiency: during the first charge–discharge process, the silicon-based anode material should minimize the irreversible consumption of lithium ions and improve the initial coulombic efficiency. (5) Good processability: the ideal silicon-based anode material should be easy to process into an electrode and be adaptable to the existing lithium-ion battery production process. (6) High safety performance: the silicon-based anode material should have good thermal and chemical stability. During the battery's charge–discharge process and under different usage environments (such as high temperature, over-charging, over-discharging, *etc.*), it should not experience thermal runaway, combustion, explosion, or other safety accidents.

To promote the commercial application of silicon-based anodes, some progress has been made through years of research. However, problems such as volume expansion and interface failure still exist during the charge–discharge process. Improvement measures such as nanostructuring, composite materials, the development of silicon-containing precursors, and prelithiation technology have alleviated these problems to a certain extent and improved the material performance. In the future, further optimization of material structure and performance should be carried out, including in-depth research on the synergistic effects of nanostructures, strengthening research on the interfaces of composite materials, improving the conversion mechanism of silicon-containing precursors, and prelithiation technology. With technological progress and cost reduction, silicon-based anode materials are expected to be more widely used in fields such as electric vehicles, energy storage systems, and aerospace.

## Data availability

No primary research results, software or code have been included and no new data were generated or analysed as part of this review.

## Author contributions

Zhenjun Zhang: writing – original draft, data curation, formal analysis. Yilong Wu: formal analysis, investigation, validation, writing – review & editing, supervision. ZuXue Mo: data curation, writing – review & editing, conceptualization. Xiaoxu Lei: visualization, validation, writing – review & editing. Xuerui Xie: supervision, writing – review & editing. Xiangyong Xue: supervision, writing – review & editing. Haiqing Qin: supervision, funding acquisition, writing – review & editing, project administration. Haowen Jiang: formal analysis, investigation, writing – review & editing, supervision, resources, methodology.

## Conflicts of interest

There are no conflicts of interest to declare.
